# Emerging Materials for Durable and Sustainable Design of Aeronautic Structures

**DOI:** 10.3390/ma18214922

**Published:** 2025-10-28

**Authors:** Pedro Carvalho, João Aguiar-Branco, Rui Miranda Guedes

**Affiliations:** 1 Department of Mechanical Engineering, Faculty of Engineering, University of Porto, Rua Dr Roberto Frias, 4200-465 Porto, Portugal; up201907099@edu.fe.up.pt (P.C.); up201907061@edu.fe.up.pt (J.A.-B.); 2 INEGI-Instituto de Engenharia Mecânica e Gestão Industrial, Rua Dr Roberto Frias, 4200-465 Porto, Portugal

**Keywords:** emerging materials, nano-materials, CFRP, life cycle assessment (LCA), recycled materials, sustainable aviation, thermoplastic composites, bio-composites, vitrimers, carbon fiber recycling, aerospace engineering

## Abstract

Sustainable and durable materials are in increasing demand as the aerospace sector seeks to reduce its environmental footprint while enhancing performance and safety. Biocomposites, recycled materials, nanomaterials, and advanced composites are being explored as alternatives to conventional aircraft materials. This work analyses the available options by comparing the mechanical properties, environmental impact, and lifecycle costs of these materials, as well as the associated manufacturing and implementation challenges. There are a few examples of next-generation materials being used in the aircraft industry. Furthermore, regulatory and technical barriers to implementation emphasize the importance of certification processes and scalability considerations. The final part explores the next generation of recyclable and sustainable composite materials, which could potentially reduce the aerospace sector’s impact on greenhouse gas emissions. These comprise future research pathways in advanced aerospace materials that will help lead the industry towards sustainability.

## 1. Introduction

Amid growing environmental concerns, the aerospace sector is struggling to address sustainability issues. As the aviation industry continues to grow, it is crucial to achieve the carbon emission reduction targets set by IATA and ICAO for 2050. One key way to accomplish this is to use lightweight, durable materials. This step will improve fuel efficiency and reduce emissions [1]. Therefore, material choice is crucial and significantly influences the performance, operating costs, and environmental impact of an aircraft throughout its lifespan [2].

In this study, “emerging materials” are defined as materials whose application in the aerospace industry is either recent, rapidly evolving, or undergoing significant technological changes, particularly in terms of sustainability, recyclability, and manufacturing processes. This classification encompasses, among others, advanced thermoplastics and bio-composites, which are being actively researched and developed as alternatives or supplements to traditional aerospace materials.

Traditional aerospace materials, such as aluminum and titanium, have long been valued for their strength-to-weight ratio, corrosion resistance, and fatigue life [3]. Recent developments in composite materials, bio-composites, and recovered metals have introduced substitutes with potential financial and environmental benefits. For instance, although advanced carbon fiber composites significantly reduce weight and improve fuel efficiency, bio-composites and thermoplastics offer better recyclability [4,5].

This study examines the development of sustainable materials for aircraft structures, with a focus on their mechanical properties. It thoroughly examines the compromises involved in using such materials in the manufacture of aircraft. The study compares conventional materials with emerging alternatives, including bio-based materials. The research also addresses the key factors in achieving a more sustainable aviation industry, as well as the technological, legal, and financial constraints involved in scaling up the use of these materials.

The conducted survey has shown that the more mature emerging solution is the replacement of thermoset resins with thermoplastic carbon fiber reinforced structures, which are undergoing intensive testing of real-scale fuselage prototypes by the aeronautics industry. Thermoplastic Carbon Fiber-Reinforced Polymers present several key advantages, in addition to their recyclability, including faster assembly through welding, improved impact resistance, and the direct incorporation of integrating systems during manufacturing. Another emerging approach being attempted is to replace the thermoset oil-based resins with bio-based resins for the matrices and to transition to bio-based carbon fibers. These technologies are not yet mature for large-scale production, nor have their mechanical performance met the requirements for the aeronautical sector. Research into the adoption of sustainable materials in the aerospace industry involves systematically comparing the life-cycle assessments (LCAs) of potential new aviation materials. The findings could inform future design decisions and legal policies, facilitating the industry’s transition to more environmentally friendly and financially viable solutions [3].

## 2. Methods

A literature survey was conducted using Scopus, ScienceDirect, and Google Scholar databases. The guidelines of this review attempt to answer the following question: What new, emerging material solutions have been studied to replace traditional materials like aluminum and titanium alloys, or the not-so-old polymer-based, reinforced polymers using carbon and glass fibers? These are interrelated with the quest for improved efficiency, sustainability, recyclability, and reduction of greenhouse gas emissions.

The scope of this review was more focused on polymer-based composites and attempts to reduce their environmental impact when applied to the transportation industry, specifically the aviation sector.

**Figure 1:**
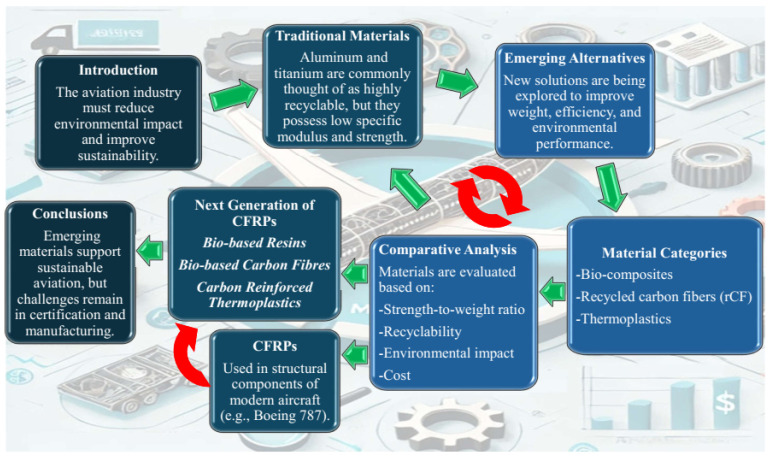
Flow chart of the study.

Figure 1 summarises the logical structure and thematic development of the article. Organised in the form of a flowchart, it comprises a series of boxes representing the core stages of the study’s reasoning.

## 3. Background on Aeronautic Material Requirements

Aerospace engineering requires careful material selection to meet safety, efficiency, and sustainability standards. Aerospace constructions greatly benefit from lightweight materials with high strength-to-weight ratios, such as aluminum, titanium, and magnesium alloys. Aluminum alloys, especially those containing lithium and zinc, have long been preferred for airplane components due to their high mechanical strength and low density [1,4]. This property not only allows for significant weight reduction, immediately translating to greater fuel efficiency and increased payload, but also fits with the industry’s desire for cost efficiency and extended service life [1,3].

The environmental and operational conditions faced by aircraft demand materials with high corrosion and fatigue resistance. Titanium alloys, renowned for their exceptional resistance to corrosion and high temperatures, are crucial in high-stress applications such as engines and other load-bearing components [2,5]. Polymer matrix composites, particularly carbon fiber-reinforced polymers (CFRPs), have also gained influence in aerospace structures due to their inherent resistance to fatigue and corrosion [1,3]. While composites reduce the impact of corrosion and offer substantial weight savings, they come with unique challenges, such as sensitivity to ultraviolet light, potential impact-related delamination, and a need for improved interlaminar strength to ensure durability under stress [1,3].

The aerospace sector also requires materials that can maintain their integrity at high temperatures, especially within engine components exposed to extreme thermal and mechanical stress. Nickel-based superalloys and ceramic matrix composites have become essential in turbine and engine sections, where temperatures often exceed 700 °C [4,5]. These materials, noted for their heat resistance and stability, contribute to reduced maintenance costs and allow engines to operate reliably under severe conditions [3,4].

The trend toward advanced composites and material innovation addresses the dual objectives of reducing aircraft weight while maintaining or even enhancing mechanical strength. Emerging materials, such as carbon nanotube-enhanced composites and ceramic matrix composites, present promising alternatives, offering high mechanical properties suitable for critical parts like fuselage and wing structures [1,2]. Nevertheless, integrating these materials into aircraft design introduces challenges, notably in terms of manufacturing complexity, cost, and the need for enhanced interlaminar strength to prevent potential delamination under load [3,5].

A key consideration in today’s aerospace material development is environmental impact. Recent research focuses on creating bio-based resins and recyclable composites to minimize the environmental footprint of aerospace materials, especially concerning end-of-life disposal [3,5]. While promising, the challenge remains in scaling these sustainable materials to meet industrial performance and regulatory standards without compromising mechanical properties [2,4]. A detailed analysis is given later.

Aerospace materials must also conform to rigorous regulatory and safety standards. Governing bodies like the Federal Aviation Administration (FAA) and European Union Aviation Safety Agency (EASA) require that all materials used in aircraft manufacturing meet specific criteria for mechanical performance and safety [1,3]. Thus, critical structural components, including the fuselage, wings, and empennages, are designed with materials that can withstand dynamic loads, pressure variations, and environmental exposure during extended service life, ensuring both operational safety and compliance with industry regulations [3,5].

Carbon Fiber Reinforced Plastics (CFRPs) enable weight reduction while maintaining the structural integrity required for aerospace applications. However, manufacturing and applying these materials bring new challenges to the industry. The durability, manufacturing technologies, and long-term performance remain complex obstacles when implementing these materials. Even though CFRPs are highly durable and lightweight, they are prone to presenting micro-cracks, delamination, and fiber-matrix separation under repeated loading or impact. These phenomena are especially concerning in the aerospace industry, as components are subjected to extreme loads in repeated cycles, and CFRPs can lose structural integrity under these conditions [6]. Likewise, bio-composites are quite sensitive to the environment in which they’re deployed, with the fibers absorbing moisture, which leads to potential swelling and weakening of the fibers, similar to the behavior of CFRPs. Ultraviolet (UV) radiation exposure, moisture, and temperature extremes can impact the long-term performance of these materials. The industry addresses these issues by focusing on fiber treatments and hybrid material solutions that enhance stability without compromising biodegradability [7].

Manufacturing CFRPs is a resource- and labor-intensive process. The complex layup and curing processes required to manufacture high-quality CFRPs are time-consuming and necessitate skilled labor, making the entire process economically challenging. The economic factor means that the application of CFRPs in large-scale industries remains challenging, resulting in their primary use in high-performance applications. Cost factors still restrict their use in commercial aviation [8]. These CFRPs also present economic disadvantages when they reach the end of their life or require repair, as repairing or recycling them is very difficult due to the need to separate the fiber from the matrix material. Techniques such as pyrolysis and solvolysis are promising but require a significant amount of energy and specialized facilities, which limit their economic feasibility for widespread adoption. For this reason, CFRPs produced using virgin carbon fibers (vCFRPs) are still the preferred material for structural components, as it is possible to have greater confidence in their mechanical properties than those produced with recycled carbon fibers (rCFRPs) [9].

The regulatory and certification processes in industries such as automotive and aerospace are stringent to ensure the safety of everyone involved. Materials must then undergo rigorous testing to assess their safety, performance, fire resistance, impact tolerance, and fatigue resistance. These tests vary depending on the criticality of the aircraft part, being more stringent for the structural components. These tests, however, lack universally accepted standards for composite processing, making certification a barrier to adoption, particularly for innovative materials that differ from traditional metals [10]. To facilitate the broader adoption of these materials, there is a growing call for updated certification standards that reflect the evolving landscape of aerospace materials. Developing regulatory frameworks that address the unique properties of CFRPs, bio-composites, thermoplastics, and rCFRPs could streamline the approval process without compromising safety. Later, we will revisit this topic to discuss it in detail.

## 4. Sustainable Materials in Aerospace

The drive towards sustainability in the aerospace sector is closely tied to the adoption of innovative materials with reduced environmental impact throughout their lifecycle. Examples include thermoplastics, bio-based carbon fiber, recycled carbon fiber, and biopolymers, as well as other materials optimized for lifecycle efficiency. To achieve net-zero emissions by 2050, the use of these materials should help reduce emissions, waste, and energy consumption [11].

In aerospace, green life cycle management (GLCM) emphasizes a comprehensive strategy that evaluates environmental impacts throughout all phases—from material sourcing to end-of-life disposal. GLCM solves the design, development, operation, and decommissioning phases, ensuring that each stage minimizes environmental impact. Emphasizing carbon emissions, energy usage, and resource depletion to pinpoint areas for development and support environmentally friendly materials, life cycle assessments (LCAs) are essential in assessing these effects [12]. A new, simplified LCA structure has been created specifically for the aerospace industry to reduce data collection complexity and enhance decision-making accuracy. This method is beneficial in evaluating sustainable materials for various uses in aerospace so that businesses may rapidly evaluate decarbonization technologies free from the significant resource requirements of standard LCA methods. The structure emphasizes the need to interact with stakeholders so that choices complement more general industry objectives for sustainability [11].

### 4.1. Bio-Composites

Bio-composites have been gaining traction in the aviation industry. These natural fibers, such as flax, hemp, or ramie, are primarily deployed within a bio-based or thermoset polymer matrix in aircraft interiors and secondary structures. The integration of these materials in aircraft interiors, such as seat panels and cabin components, has demonstrated significant potential for reducing the carbon footprint associated with the production of these parts. A lifecycle assessment (LCA) indicated that using bio-composites instead of traditional materials could reduce the carbon footprint and energy consumption by 38%. However, this decrease in carbon footprint comes with an increase in water consumption of approximately 45% [13].

Bio-composites present significant advantages and opportunities over traditional materials. These materials have lower density, higher biodegradability, and a reduced cost compared to an equivalent conventional material. However, their lower density doesn’t compromise their structural integrity [14]. Especially in the aeronautical industry, a higher material efficiency is particularly relevant, as it directly impacts the payload and range of the aircraft. Manufacturing these materials is also less hazardous and requires less skilled labor than traditional materials [15].

Analysis of lifecycles highlights the environmental advantages of bio-composites in aviation. One comparative research study found that they had a minor environmental impact compared to conventional composites, particularly in end-of-life situations, when bio-composite laminates can be composted or recycled more effectively than synthetic composites. Because bio-composites reduce weight and translate into lower fuel use, they can also help to save operating costs. For example, studies on bio-composites for airplane interiors found that, with less weight, one business-class seat produced might save an airline over €3382 over five years of use [13].

Despite all the advantages mentioned before, bio-materials also present some challenges for implementation. Combining natural fibers like flax and hemp in a polymer matrix, bio-composites provide clear environmental benefits. However, they have natural limits in mechanical characteristics. Flammability and moisture sensitivity remain significant challenges for these materials. Natural fibers contain cellulose, which decomposes at relatively low temperatures, releasing flammable gases that compromise fire safety in aviation. A review of natural fiber flammability indicates that cellulose-based fibers, such as flax and hemp, are susceptible to thermal decomposition, releasing combustible volatiles that reduce their suitability for load-bearing applications in high-temperature conditions. This limitation has led to research into chemical treatments and flame retardants to enhance the fire resistance of bio-composites, though these treatments may affect the mechanical properties [16]. Even with chemical treatments, bio-composites struggle to meet the strict fire safety criteria required for structural components and aircraft interiors [11].

Studies were conducted on a bio-based resin matrix and a rosin-based epoxy, incorporating two different stacking arrangements into a carbon fibre reinforced polymer. The results of the vertical burning test indicated that all samples passed according to FAR 25.853. However, mechanical testing revealed a reduction in the tensile and flexural properties of the hybrid laminates due to the FR mats [17]. The mechanical properties of biocomposites are significantly enhanced by incorporating hybrid natural fibres. However, single biocomposites were more impact-resistant than hybrid biocomposites. Nevertheless, hybrid biocomposites exhibited the highest thermal stability, with the highest initial and final decomposition temperatures [18]. Another study found that epoxy-reinforced flax fibre treated with two types of flame retardant met the burn length requirements set out in FAR 25.853 for use in aircraft interior components. Both the treated and untreated flax fibre composites were successful. Although flax fibre composites show promise in reducing flammability and limiting toxic gas emissions, particularly when treated with flame retardants, concerns remain regarding the performance of the treated composites in terms of smoke density and heat release [19].

Natural fibers are inherently hydrophilic, which can lead to potential delamination and decreased mechanical strength over time when exposed to moisture. Moisture can damage the mechanical properties of bio-composites, causing delamination and loss of stiffness and strength. Natural fibers, such as flax, can absorb moisture, leading to dimensional instability and mechanical degradation over time. For example, flax and hemp fibers, commonly used in bio-composites, can absorb significant amounts of humidity, leading to structural instability [20]. Some treatments, such as Alkali treatments, have been used to reduce water absorption and improve the compatibility of these materials with application in the aeronautics industry [14]. Hybridization with synthetic fibers and surface treatments can help offset these problems somewhat, but this approach complicates manufacturing and increases expense [11].

The ECO-COMPASS EU/China project identified improvements needed in the performance of such materials concerning moisture ingress, fire ignition and propagation, creep, and ageing. An innovative approach to each type of natural fibre and resin system may help tackle these drawbacks. To optimise the performance of eco-composites, the positive features of bio-based materials, such as the low density and noise-reduction properties of natural fibres, can be further exploited [21].

The literature proposes integrating fibres such as flax, hemp, and ramie into a bio-based or thermoset polymer matrix for use primarily in aircraft interiors and secondary structures, including seat panels and cabin components. However, the mechanical performance of these composites does not match that of aerospace-grade carbon fibre reinforced plastics (CFRPs). Their properties are also significantly affected by after-hygrothermal ageing as shown in Table 1.

Although manufacturing bio-composites is often less expensive than synthetic substitutes, consistency in quality is difficult. Standardizing a material becomes challenging when natural elements can cause variations in fiber characteristics, which in turn influence its mechanical performance. Higher scrap rates and more expenses in quality control resulting from this fluctuation might discourage producers. The inherent variability of natural fibres makes it difficult to ensure consistent quality that meets aerospace standards, necessitating intensive processing. It highlights the importance of thorough processing to fulfil aircraft criteria [11].

Strong safety and performance criteria make achieving regulatory certification for bio-composites in aeronautical uses challenging. As the certification process in the aerospace industry is rigorous, materials must consistently meet stringent standards of strength, durability, and flame resistance. Bio-composites face regulatory challenges due to limited data on long-term performance and property consistency; therefore, certification becomes a time-consuming and costly procedure [11]. All things considered, bio-composites show promise as a sustainable substitute for various aerospace applications. Although issues with flammability and moisture absorption are significant barriers to broader use, they provide operational and environmental benefits, especially in non-load-bearing structures. Expanding bio-composites in the aviation sector would depend critically on improving material treatment and reinforcing methods.

**Figure 2:**
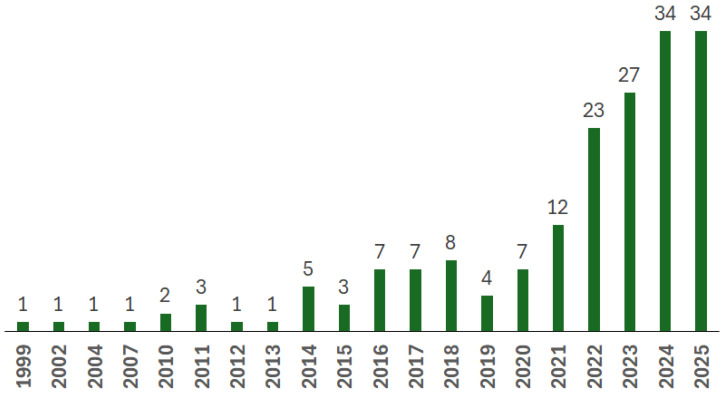
Number of publications about bio-based composites per year.

Figure 2 shows the results of a survey on the number of research or review articles published in journals or presented at conferences using the keyword ‘biocomposites’, restricted to cases applicable to the aerospace and aircraft industry. Publicly available research in this field is still in its infancy.

### 4.2. Recycled Carbon Fiber

To further improve sustainability in the aeronautics sector, the manufacturing processes for new materials must be optimized, and the recyclability of existing materials must also be improved. Some modern materials are challenging to recycle; however, as carbon fiber-reinforced composites reach the end of their life, their recyclability has become a significant focus point due to their increasing demand and use in aircraft. Several techniques, including mechanical recycling, pyrolysis, and solvolysis, have already been implemented and are continually being improved.

A significant advantage of recycled carbon fiber (rCF) is the much lower energy needed for its manufacturing. While virgin carbon fibers (vCF) require energy-intensive manufacturing processes costing over 290 MJ/kg, the recycled variant only requires about 10% of that [27,28]. Studies show that keeping resources at their highest value possible, by using composites reinforced with rCFs (rCFRPs) in non-structural applications, can help reduce carbon emissions, decrease landfill waste, and promote a circular economy [27].

Among the available recycling methods for CFRP waste, mechanical recycling, which involves grinding or milling the material into tiny particles, stands out mainly for its low cost. However, this method inherently produces discontinuous fibers, significantly reducing their mechanical properties, especially tensile strength, with a loss of up to 50% compared to the original fibers [29]. Mechanically recycled carbon fibers retain only about 70% of the tensile strength of virgin fibers, rendering them less appropriate for load-bearing uses in aircraft. Due to these limitations, mechanically recycled CFRPs are restricted to non-structural or secondary applications. Recent advances, such as electrodynamic fragmentation, have shown promise as an alternative to traditional grinding. This technique aims to reduce fiber damage, potentially preserving up to 83% of the tensile strength, which could expand the use of recycled fibers into structural applications, including those in the aerospace sector [28].

Pyrolysis is another recycling method often used. In this method, resin is broken down at high temperatures in an inert environment, thereby protecting the carbon fibers and facilitating resin decomposition. The thermal stability of rCFRPs obtained by this method qualifies them for many aerospace parts. However, residual char sometimes adheres to the fibers; hence, post-treatment is necessary for optimal performance. These additional steps add complexity, cost, and time, making mechanical grinding less economically appealing than [27]. These fibers can yield up to 80% of the tensile strength of virgin carbon fibers, making them suitable for use in non-critical aerospace applications [29].

Fiber length is another critical aspect. Often emerging as short filaments, mechanically regenerated carbon fibers have little reinforcing capacity. Shortened fiber length from mechanical recycling results in weaker bonding within the polymer matrix, thereby restricting the material’s load-bearing capabilities. Improved alignment and impregnation techniques are being developed to optimize rCF for broader aerospace applications [27]. Recycled fibers require up to 90% less energy during manufacturing than new carbon fiber, significantly reducing their environmental impact. Recent research has shown that advances in alignment techniques and fiber treatment enhance the mechanical properties of recycled fibers, thereby broadening their applicability within the sector [12].

Combining bio-based materials and recycled carbon fiber effectively demonstrates the shift toward sustainable, circular materials in aircraft. Bio-based composites are designed using renewable fibers, such as hemp and flax, which have reduced carbon emissions during manufacture and higher biodegradability. They have difficulties in flammability and moisture resistance, limiting their applicability to interior and non-load-bearing structures. An LCA research revealed that bio-composites might lower lifetime emissions by up to 40% compared to conventional composites [12].

Although they have significant environmental advantages, recycled carbon fiber composites lack fiber strength and integrity. While promising, rCF struggles with fiber integrity, contamination, and restricted mechanical qualities compared to vCF. The solvolysis process, which dissolves the resin using supercritical fluids, offers an advantage by maintaining fiber strength close to that of virgin carbon fibers. However, as Chaanai et al. note [30], high prices, operational complexity, and the need for large volumes of solvents—which generate environmental disposal issues—limit the use of solvolysis. Furthermore, an environmental evaluation study reported that “solvolysis presents unique challenges, including fiber diameter variations and char residue, which impact the quality and consistency of rCF for aerospace applications” [29]. The cost of solvolysis equipment and the need for large quantities of solvents restrict the scalability of rCFRPs, limiting the cost-effectiveness of their widespread adoption in aerospace [12].

Limited recycling infrastructure and the complex nature of composite waste streams pose logistical difficulties for scaling up rCFRP manufacture. Further complicating the recycling process are fiber contamination and matrix residue from the recycling process, which necessitate additional cleaning phases, thereby increasing manufacturing expenses. The high cost of pyrolysis and the limited recycling infrastructure make it difficult for aerospace manufacturers to incorporate rCFRP on a large scale.

Recent recycling approaches that preserve fibre architecture have been shown to significantly maintain the mechanical reinforcement capability of recycled carbon fibre (rCF). Preserving woven fibre architectures is more feasible than preserving unidirectional fibres, since the latter require careful handling throughout the entire process, thereby limiting high-volume recycling. A comparison of the mechanical properties of virgin and recycled carbon fibres is given in Table 2, obtained from [31].

Recycled CFs have essential environmental advantages by reducing the demand for virgin carbon fiber manufacture and cutting energy usage and greenhouse gas emissions. Recycled CFRP components provide substantial energy savings, contributing positively to circular economy goals by diverting composite waste from landfills [29]. Including rCF into the supply chain offers a workable way to lower carbon footprints, reduce dependency on limited resources, and lower manufacturing expenses. A modern assessment highlighted the potential of recycled carbon fiber in reducing total manufacturing costs, thereby promoting greater use in general aviation [27].

Recycled carbon fiber composites also face regulatory hurdles due to inconsistent performance data. Obtaining approval for structural uses is challenging, as recycled fibers often fail to meet the stringent criteria for durability and strength established by aircraft authorities. Extensive testing is part of the regulatory procedure to show that recycled composites can consistently operate in safety-critical parts. Comparatively, the variability in fiber quality from recycled sources complicates certification, as regulatory agencies require proof of consistent mechanical properties [11]. Such certification requirements limit the acceptance of rCFRP in main aircraft structures by increasing time and expense.

**Figure 3:**
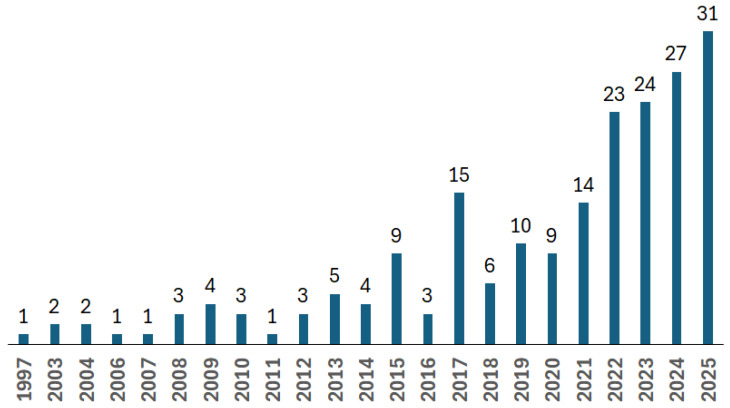
Number of publications about recycled carbon fibres per year.

Figure 3 illustrates the evolution of publications on recycled carbon fibres in the aerospace and aviation sectors. These articles have either been published in journals or presented at conferences. Growth in publicly available research in this field is moderate. The limited number of publications may be related to the fact that current solutions cannot produce recycled fibres that meet the requirements of the aerospace industry.

### 4.3. High-Performance Thermoplastic Composites

Thermoplastic composites, known for their recyclability, durability, and efficient processing, are emerging as viable alternatives to traditional thermoset composites in aerospace applications. The aerospace sector is increasingly favoring thermoplastic polymers like polyether-ether-ketone (PEEK), polyether-ketone-ketone (PEKK), polyphenylene sulfide (PPS), and poly(phenylene sulfide sulphone) (PPSS) due to their high-temperature resistance, mechanical strength, and potential for cost-effective processing [32,33]. Using thermoplastic composites may reduce energy needs in manufacturing by up to 25%, according to studies, thereby helping to lower greenhouse gas emissions during the material’s lifetime [12].

Because of their recyclability, repairability, and shorter manufacturing times, thermoplastic composites are gaining growing acceptance. They can be heated and reshaped, unlike thermosets, enabling more straightforward repairs and less waste. These composites are suitable for applications requiring high impact resistance, chemical stability, and heat tolerance. It makes them practical in primary and supplementary aircraft structures, especially when durability and weight reduction are critical [12].

Additionally, PEEK and PPS have demonstrated excellent mechanical properties, with PEEK offering high fracture toughness and tensile strength, which supports its application in load-bearing aerospace structures. Comparative studies of PPS and PEEK matrices indicate that PEEK possesses superior fracture toughness, with values 4–8 times greater than those of PPS, thereby enhancing damage tolerance in structural components [34]. This increased toughness enhances resistance to crack propagation and delamination under impact, making PEEK an ideal material for components subjected to high stress and repeated loading.

Thermoplastic composites can be manufactured rapidly without the use of autoclaves, unlike thermosets, which reduces production costs and energy consumption. A study on processing efficiencies noted that thermoplastics, such as PEEK and PPS, can be consolidated in minutes, compared to the extensive curing cycles required for thermoset resins [35]. This streamlined processing reduces production time and further contributes to cost efficiency. Furthermore, thermoplastic composites do not require refrigeration, simplifying storage and transport and reducing lifecycle costs [33]. Their ability to be reshaped and repaired via thermal processes means that damaged parts can often be refurbished rather than replaced. For example, PPS and PEEK composites can undergo welding and reshaping, minimizing waste and enhancing recyclability [36].

Despite these advantages, thermoplastics face challenges with processing temperatures and material costs. PEEK, for instance, requires processing temperatures exceeding 370 °C, necessitating specialized tooling and machinery, which increases upfront investment [37]. Additionally, although PPS is relatively more affordable and processes at lower temperatures, it lacks PEEK’s high tensile strength and fracture toughness, limiting its application in critical load-bearing components. Moreover, the stiffness of thermoplastic prepregs poses layup challenges, particularly for complex geometries. A study on high-performance thermoplastic composites reported that “thermoplastics are less pliable than thermosets, making them less suitable for hand layup processes, particularly in intricate or contoured designs” [35]. This characteristic limits their use in highly contoured aerospace components, though automated fiber placement (AFP) and automated tape laying (ATL) technologies are making strides in overcoming these limitations [37].

The increased viscosity of thermoplastic composites exacerbates fiber impregnation, often resulting in voids within the composite construction and compromising its mechanical strength. Studies on thermoplastic laminates show that the high viscosity of thermoplastic resins makes achieving complete impregnation difficult, often resulting in void content, which weakens the final composite [12]. This restriction influences the performance of the composite in load-bearing aircraft components, where homogeneity and strength are vital.

High-performance thermoplastics like PEEK and PPS are expensive; therefore, their use is limited to those who can justify the additional cost based on their advantages. Furthermore, adding to capital expenses are specific tools required to handle these materials. Thermoplastic composites are more expensive than their thermoset counterparts due to the higher material costs and the advanced machinery needed for high-temperature processing [12].

Although thermoplastic composites are more readily recyclable than thermosets, the technique still has financial challenges. In many cases, the recycling process of several plastics yields a phase-separate mixture, necessitating the use of compatibilizers to control phase separation, which increases the cost and reduces profitability. These difficulties partly explain the low recycling rate of thermoplastics that have been consumed. Moreover, current recycling techniques focus on thermoplastics, omitting thermoset plastics, which account for one-third of the total plastic manufactured. These materials have rigid cross-linking, which makes them difficult to recycle and often results in their mechanical grinding into powder for use in lower-grade applications [38]. Despite the claimed recyclability of thermoplastics, traditional recycling processes have been applied to both thermosetting and thermoplastic composites, ultimately producing downgraded materials. The status of these end-of-life processes and future trends was recently extensively analyzed [39,40].

Like rCFRP, thermoplastic composites face regulatory challenges primarily related to variations in characteristics that depend on production conditions. While PEEK and PPS composites must consistently perform under high stress and temperature, material dependability may be affected by variance in fiber-matrix bonding brought on by high processing temperatures. The time and money required for certification are further compounded by regulatory agencies’ sometimes-demanding requirements for extensive testing to ensure that thermoplastic composites meet durability and thermal resistance standards [12].

**Figure 4:**
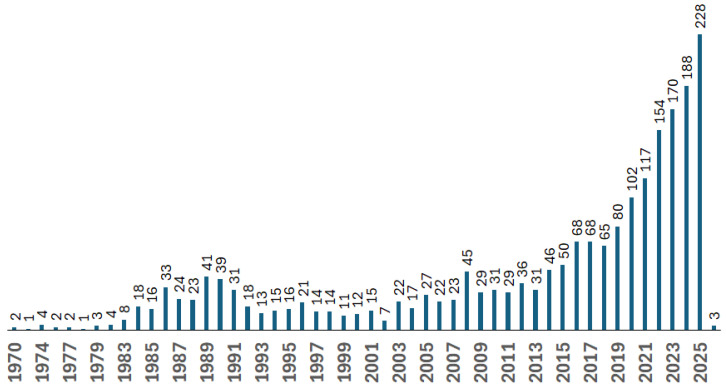
Number of publications about thermoplastic composites per year.

Figure 4 illustrates the evolution of publications on thermoplastic composites applicable to the aerospace and aircraft industries. These publications have been either published in journals or presented at conferences. Publicly available research in this field is growing moderately. It may be because companies are restricting the release of sensitive information that is critical to maintaining their competitiveness.

### 4.4. Epoxy Vitrimers and Composites

Repair of thermoset epoxy polymers and composites using welding or applying heat is not feasible because the material undergoes an irreversible chemical reaction during curing and cannot be melted. The solution to this issue, which enables recyclability, leads to a new category of thermoset epoxy resins, known as vitrimers [41]. The breakthrough occurred in 2011, with a source-based recovery strategy, as described by Leibler et al. [42]. The permanent crosslinks are replaced with exchangeable bonds that can rearrange thermally via dissociative or associative mechanisms. Above the topology freezing temperature, these bonds can rearrange to repair resin/fiber delaminations and resin microcracks in the damaged part. The topology-freezing temperature is a key physical property of vitrimers, changing between a solid-like and a liquid-like state. This effect is caused by the activation and deactivation of bond exchange in the network. Nevertheless, bond exchange may occur locally at temperatures well below this transition temperature, at which point vitrimer-specific properties, such as relaxation and creep, become apparent. Crucially, vitrimers maintain the properties of traditional thermosets when operating at high temperatures. These properties include resistance to environmental degradation, excellent thermal performance, and high mechanical strength [43,44]. Recently, a new aerospace-grade vitrimer (3R), formulated from a mixture of the monomers Tetraglycidyl-4,40 methylene dianiline (TGMDA) and Bisphenol F diglycidyl ether (BFDGE), and the hardener 4-Aminophenyl disulfide (4-AFD), was compared with a conventional, typical aerospace-grade epoxy resin (RTM6). Both resins were used to produce laminated CFRP using a ${\left[45/0/90\right]}_{4}s$ lay-up sequence. The comparison was done via knockdown factors. The interlaminar shear strength test (ILSS) and low-velocity impact results demonstrated negligible knockdown factors, with values below 4%. The repair efficiency for the ILSS geometry was 72%. At low-velocity impact specimens, the repair efficiency was 87% for the average maximum load and 105% for the absorbed energy. In the lap strap geometry, the knockdown factor is 3% from RTM6 to 3R composites. However, the 3R lap strap specimens recovered approximately 110% of their initial values after welding during the repair process [45].

Although epoxy vitrimers have proven to enhance the sustainability of composite materials, significant challenges arise when transitioning from lab-scale concepts to real-world applications. There is a need to increase the glass transition temperature to meet the structural application requirements. Furthermore, large-scale production requires the use of affordable catalysts to be sustainable [46,47].

## 5. Comparative Analysis of Materials Performance

In aerospace engineering, material selection is crucial for balancing structural integrity, weight, and sustainability. Traditional materials, notably aluminum and titanium alloys, have long been favored for their durability and established manufacturing processes. However, composite materials, particularly carbon fiber-reinforced polymers and ceramic matrix composites, offer significant advantages in weight reduction, fuel efficiency, and emissions reduction over an aircraft’s lifecycle. This comparative analysis examines the environmental impact, lifecycle efficiency, and practical challenges associated with these materials in the aerospace industry.

### 5.1. Material Performance and Strength-to-Weight Ratio

The performance of aerospace materials is determined mainly by their strength-to-weight ratio, which significantly impacts fuel efficiency, payload capacity, and overall operational costs. Traditional materials, like aluminum alloys (notably the 2xxx and 7xxx series), have long been used in aircraft structures due to their balanced mechanical properties, cost-effectiveness, and well-established manufacturing processes [48]. Aluminum is particularly valued for its tensile strength and resistance to corrosion, especially in fuselage and wing structures. However, aluminum alloys are denser than advanced composites, which can limit the weight reduction achievable in highly optimized aircraft designs [49].

Carbon fiber-reinforced polymers offer a significantly higher strength-to-weight ratio than traditional metals. CFRPs, which are approximately 20–30% lighter than aluminum, allow for substantial weight reductions. This weight advantage directly affects fuel consumption, as lighter aircraft require less thrust to maintain flight, leading to fuel savings of up to 20% over the aircraft’s lifespan [48,50]. Moreover, CFRPs’ stiffness-to-weight ratio enables them to maintain structural rigidity while reducing weight, which is essential for load-bearing components such as wings and fuselage skins. This property enabled the use of CFRP in high-profile aircraft models, such as the Boeing 787 and Airbus A350, where over 50% of the structural material by weight is composite, primarily CFRP [51].

### 5.2. Performance in High-Stress and High-Temperature Applications

Titanium alloys, while denser than both aluminum and CFRP, provide an ideal balance of high strength, corrosion resistance, and temperature resilience, making them suitable for components subjected to extreme stress and temperature fluctuations, such as engine casings and landing gear [49]. Titanium has a density of around 60% of aluminium’s, yet it is stronger and more durable under extreme thermal conditions. It has a maximum operating temperature of 600 °C in engine applications. Using titanium in critical areas enables manufacturers to maintain safety margins without substantially increasing weight. However, it should be noted that the extraction and processing of titanium remains costly [2].

Ceramic matrix composites (CMCs) represent an emerging material group that excels in high-temperature applications. Unlike metal alloys, CMCs can withstand temperatures exceeding 1000 °C, making them ideal for next-generation turbine engines, where they enhance thermal efficiency and reduce cooling requirements. These composites enable engine components to operate at higher temperatures, resulting in improved fuel efficiency. CMCs, however, face manufacturing challenges and are generally restricted to specific, high-cost applications due to their brittle nature and expensive production processes [48,51].

### 5.3. Fatigue and Impact Resistance

Fatigue resistance is a crucial factor in determining the performance of aerospace materials, particularly in the context of the cyclic loading experienced by aircraft. Traditional metals, such as aluminum and titanium, have well-understood fatigue behavior, with predictable failure modes that can be managed through regular inspection and maintenance. Aluminum alloys, however, are susceptible to fatigue cracking, particularly around fasteners and joints, which can limit their lifespan in specific applications [48].

In contrast, CFRPs exhibit excellent fatigue resistance due to their composite structure, which distributes stress across multiple fiber layers, reducing the likelihood of crack initiation and propagation. CFRPs also demonstrate good corrosion resistance, unlike metals, which can corrode under environmental exposure. However, CFRPs are prone to delamination (the separation of fiber layers) and are more susceptible to impact damage than metals. This sensitivity to impact is reflected in the need for specialized inspection techniques, such as ultrasonic testing, and advanced repair methods to restore their integrity, adding to the maintenance complexity [49,50].

Thermoplastic composites (TPCs) exhibit excellent fatigue resistance compared to traditional metals and even some thermoset composites. The thermoplastic matrix in TPCs provides flexibility and energy absorption capabilities, which allow these materials to withstand repeated loading cycles without significant degradation. Unlike metals, which can develop micro-cracks and fatigue fractures over time, TPCs distribute stress across multiple layers of reinforced fibers, making them less susceptible to fatigue-induced failure [48,50].

In addition to fatigue resistance, TPCs are known for their high impact resistance, as they are inherently tougher than thermosets due to their capacity to absorb and dissipate impact energy more effectively. When subjected to sudden loads or impacts, the flexible nature of the thermoplastic matrix helps prevent failure, as it enables the material to deform without breaking. This resilience to impact is crucial in applications such as interior panels, cargo doors, and secondary structural components, which may face unexpected shocks during handling or in-flight incidents [49,51]. Table 3 summarizes the fatigue and impact resistance of the primary material groups evaluated in this study, as well as their general suitability for aeronautical structural applications at the moment.

Research continues to improve the performance characteristics of CFRPs and other emerging materials. Innovations in fiber-matrix bonding are enhancing the interlaminar strength of CFRP, mitigating delamination risks, and increasing its impact resistance. Moreover, hybrid composites that combine CFRP with other materials, such as GLARE (glass-fiber-reinforced aluminum laminate), are being developed to optimize weight reduction while retaining damage tolerance and impact resistance. GLARE, already used in the Airbus A380 fuselage, combines the benefits of aluminum and composites to enhance fatigue life and safety in critical load-bearing structures [50,51]. A comprehensive comparison of key mechanical and thermal properties is provided in Table 4.

### 5.4. Cost-Efficiency

While aluminum remains the most cost-effective material for many aerospace applications, its cost efficiency is often limited to initial manufacturing and its widespread recyclability. Aluminum is relatively cheap to produce and has well-established processes for repair and recycling, which make it attractive for secondary structures and components with less critical performance demands. Due to these advantages, aluminum remains widely used in aerospace, especially in applications where weight reduction is less vital, and cost savings are prioritized [49].

However, materials like carbon fiber-reinforced polymers (CFRPs) offer significant economic benefits over an aircraft’s lifespan, especially for high-usage, fuel-sensitive applications. Although CFRP production incurs high initial costs due to complex manufacturing and curing processes, it provides substantial long-term savings through weight reduction and associated fuel efficiency. For instance, CFRP’s high strength-to-weight ratio reduces the aircraft’s weight, leading to lower fuel consumption. Over time, these fuel savings can offset the higher upfront production costs, making CFRPs economically favorable for load-bearing structures in high-use aircraft. Nevertheless, CFRPs are less cost-effective in secondary or non-load-bearing structures, where their performance advantages are less impactful, and production and repair costs are more limiting [48,51].

Thermoplastic composites, while initially more expensive than aluminum, offer a unique balance between cost-efficiency, recyclability, and ease of repair. TPCs can be reshaped or repaired when damaged, reducing maintenance costs over the aircraft’s life. This repairability is a significant advantage over CFRPs, which are more challenging and costly to repair due to their thermoset matrix. In CFRPs, damage often leads to delamination or fiber fracture, requiring complex repair techniques that may necessitate full part replacement. By contrast, TPCs can be reheated and reshaped, allowing for faster and more economical repairs. It is particularly advantageous in applications that involve heavy use, where minimal maintenance is crucial [2,51].

The recyclability of TPCs further enhances their economic value compared to both CFRPs and traditional metals. Unlike CFRPs, which require energy-intensive processes like pyrolysis or solvolysis to reclaim fibers, TPCs can be recycled by melting and reforming. This more straightforward recycling process makes this material a more sustainable and economically advantageous choice, especially for airlines focused on long-term sustainability and reducing end-of-life disposal costs. Aluminum, while highly recyclable, does not offer the same weight reduction benefits as TPCs, which can limit its economic benefits in terms of operational savings over an aircraft’s lifecycle [2,51]. Table 5 presents a comparative analysis of cost-related parameters for each mater, including production cost, repair and maintenance complexity, recyclability potential, and long-term lifecycle cost-efficiency.

## 6. Composite Materials in the Aircraft Industry and Known Issues

The aerospace sector’s commitment to improving fuel efficiency, reducing emissions, and lowering maintenance costs has led to a greater focus on composite materials, particularly carbon fiber-reinforced polymers (CFRPs). Traditional metals, such as aluminum and titanium, have been the backbone of aerospace structures due to their strength, durability, and established manufacturing processes. However, the developments of the last decade, culminating in the Boeing 787 Dreamliner, underscore the transformative potential of composites in achieving these performance goals.

**Figure 5:**
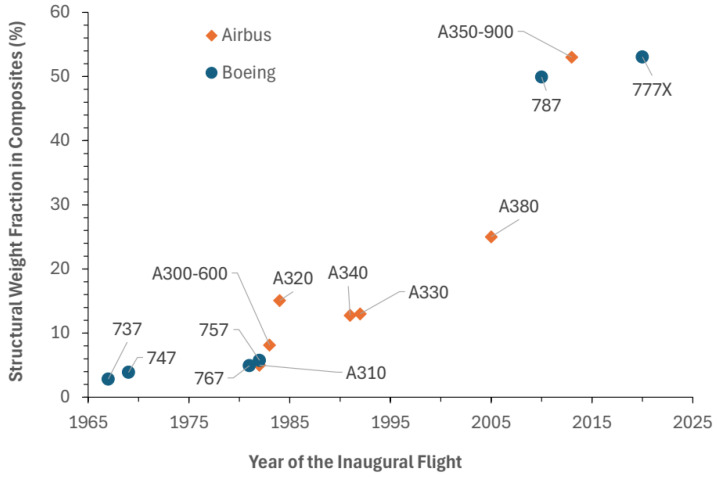
The evolution of the structural weight fraction of composite materials in the commercial aircraft sector (data collected from manufacturers).

The Boeing 787 integrates more than 50% CFRP by weight in its primary structure, including the fuselage, wings, and empennage. This design change has enabled substantial fuel efficiency gains—up to 20% over conventional aluminum-intensive designs. Such improvements are attributed to the high strength-to-weight ratio of CFRP, which allows a considerable reduction in weight while maintaining structural integrity. In turn, weight savings contribute to lower fuel consumption and emissions, making CFRPs a strategic choice to meet industry-wide sustainability goals [52]. To contextualise the industrial relevance of these materials, Figure 5 presents the evolution of the composite weight fraction in the primary structure of commercial aircraft, reaching more than 50% with the Boeing 787 Dreamliner and Airbus A350-900.

The mechanical properties of CFRP play a critical role in its application in high-load-bearing aircraft components. CFRPs offer superior tensile strength and stiffness compared to aluminum, and their fatigue resistance enables longer maintenance intervals and reduced inspection frequency. This resilience to cyclic loading is particularly beneficial in fuselage and wing structures, where CFRPs’ ability to withstand repeated stress without significant degradation extends the operational life of the components. Unlike metals, CFRP structures do not suffer from corrosion, which reduces the need for regular maintenance associated with corrosion control and mitigates the risks of weight penalties due to the use of corrosion-resistant coatings or treatments. This durability not only lowers maintenance costs but also enhances the overall economic efficiency of aircraft operations [52].

However, the production and integration of CFRPs present unique engineering challenges. The carbon fiber manufacturing process, often derived from polyacrylonitrile (PAN), is energy-intensive, involving high-temperature processing to create the carbonized fiber structures necessary for aerospace-grade composites. Additionally, CFRP components require careful handling during the layup and curing stages to avoid defects such as voids and delamination, which can compromise their structural integrity. Despite these challenges, advanced manufacturing techniques, including automated fiber placement and resin transfer molding, are enhancing process reliability and reducing production time and costs, thereby supporting the wider adoption of CFRP in aerospace applications [48,53].

The environmental impact of composite materials is another critical consideration. While CFRP manufacturing has higher initial emissions due to energy consumption, lifecycle assessments indicate that these materials have a lower environmental footprint than aluminum over the aircraft’s operational life. The fuel savings enabled by CFRP’s weight reduction result in cumulative emissions reductions that offset the initial environmental impact of manufacturing. Studies indicate that beyond specific break-even points—generally around 300,000 km of flight distance—CFRP becomes more environmentally favorable than aluminum, highlighting the material’s suitability for high-usage commercial aircraft. This lifecycle advantage aligns well with the aerospace industry’s commitment to reducing greenhouse gas emissions and supports international goals for carbon neutrality by 2050 [48,54].

From a structural performance perspective, CFRPs exhibit failure mechanisms distinct from metals, necessitating specialized design and maintenance approaches. Delamination, a primary failure mode in composites, involves the separation of fiber layers within the laminate, which can significantly reduce the load-bearing capacity. Understanding and mitigating delamination have become focal points in composite engineering [53].

Moreover, advancements in textile composites, including 3D woven and braided carbon and glass fiber reinforcements, offer additional benefits. Textile composites improve impact resistance and energy absorption, making them suitable for components subjected to high dynamic loads. These materials provide structural integrity even in complex geometries, where traditional laminate structures may struggle. For instance, 3D woven composites are increasingly being explored for applications that require enhanced damage tolerance, as they reduce the propagation of cracks and improve post-impact performance. By optimizing fiber architecture, engineers are developing composites that not only meet structural requirements but also contribute to overall weight savings, enhancing both performance and sustainability [49].

## 7. Material Qualification and Next-Generation Composites

Although promising, the integration of sustainable materials in aircraft presents significant challenges regarding performance, cost, ease of manufacturing, and regulatory acceptance. These issues must be carefully addressed for bio-composites, and thermoplastic composites to reach general acceptance.

In the aerospace industry and others, there is a need to continue reducing weight while increasing strength and durability. There is also a need for greater design flexibility and improved sustainability, i.e., reducing environmental impact. Complex shapes and integrated functionalities are often required, and composite technology is the most effective solution. In addition to these requirements, materials used in aerospace applications must withstand extreme environmental conditions while being fatigue-resistant and damage-tolerant. Thus, composites are unanimously elected to continue paving the way for the next generation of materials and structures. Although this trend is evident to everyone in the industry, many obstacles remain to reducing ${\mathrm{CO}}_{2}$ emissions and enhancing recyclability to expected levels within the aerospace sector within the next decade.

Over the last two decades, the National Aeronautics and Space Administration (NASA), the Federal Aviation Administration (FAA), industry, and universities have attempted to establish a centralized composite material property database, similar to those for metals. Still, despite advances, it has not been achieved yet [55]. The reason lies in the polymer-based composite materials paradigm, which contrasts with metals. In the case of metals, each alloy and the processes used in aircraft design are fixed and independently approved in the document, alongside an exhaustive description of the material (the Material Specification). The properties of the materials are measured experimentally under the influence of various expected in-service factors. Therefore, standard specimens fabricated according to approved processes determine the structure’s response. Since the composition of previously qualified materials remains unchanged during the fabrication of the aircraft structure, certification is completed based on the previously determined properties. In the case of composites, the adapted methodology considers the ply properties as fundamental to form a base characteristic of the material, i.e., to form the designated Building Block [56]. It works for prepreg-fabricated laminated composites in the autoclave, the typical process in the aircraft industry [57], and provides extensive material databases and methods to substantiate the equivalent properties of various materials available.

The inability of the mechanical properties of plies, as determined by coupon testing, to accurately predict the mechanical behavior of laminates becomes more pronounced in more complex fabrication processes. Therefore, as recognized in the European Space Agency (ESA) rules for large aeroplanes, AMC 25.613, simple material test coupons at the base of a typical test pyramid (Basic Block) may not accurately represent the material strength and other properties of the final part. It means that design values are determined based on the laminates of interest. Consequently, the necessary tests require considerable time and resources to cover all the laminates and thicknesses of interest [58].

Adopting rapidly new materials and fabrication methods necessitates a distinct regulatory approach. While ’material’ and ’structure’ are usually different, this distinction becomes irrelevant for complex integral composite structures formed using ’combined’ processes. These structures are formed simultaneously and are unique. Therefore, the certification approach for aircraft structures incorporating these solutions must recognize the fundamental nature of composite materials [58]. The multiscale or trans-scale approaches have been extensively developed over the past two decades [59,60,61]. It assumes the role of a methodological framework that will reduce the experimental efforts required to build and certify new composite materials [62].

**Figure 6:**
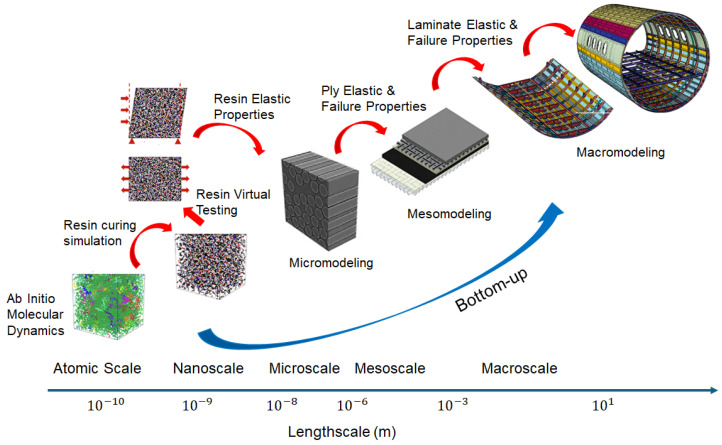
Bottom-up multiscale approach (Adapted from [67]).

A significant breakthrough was achieved by adopting a multiscale, bottom-up strategy instead of the traditional top-down approach. This strategy begins with a micromechanics analysis, proceeds to the ply stacking level of the laminate, and then considers the structural scale to capture structural failure modes [63,64]. Recently, two additional levels were added at the bottom of the scale to start from the resin-matrix design. Starting with the atomic scale, a quantum-chemical reaction path is used to calculate the reaction characteristics of epoxy curing. Then, molecular dynamics simulations are used to determine epoxy properties at the molecular scale [65]. This enhanced, multiscale, bottom-up approach predicts the mechanical performance of composite materials by designing their fundamental components, particularly the resin, as this plays a vital role in performance. The next step is to develop a method for predicting interlaminar properties since these are a key factor in delamination. The quantum-to-molecular-to-continuum bottom-up modelling should help reduce the need for testing the resin properties as a single constituent. The importance of this model concept for virtual testing is closely tied to the diversity of resin systems and the transition from thermoset to thermoplastic matrices. It would allow for a reduction in the number of physical tests required of the resin. This data, coupled with the fiber properties that comprise the level zero of the Building Block, is then used to calculate the ply properties using homogenization techniques. These contributions will help reduce the number of experiments required to verify the safety of aerospace structural parts, as illustrated in Figure 6. The European NHYTE Project investigated the accuracy of virtual testing in predicting the correct values for the design of composite structures. The project aimed to develop and characterize innovative, integrated aerospace structures made of a new hybrid thermoplastic matrix composite material with multifunctional capabilities. They employed a micromechanics model that considers the various basic components (PEEK matrix, PEI films, and fibre reinforcement) to adopt a multiscale approach, ultimately reaching the coupon level. Therefore, the coupons could be tested virtually. The reported case [66], which does not include the quantum-to-molecular-to-continuum modeling of the matrix resin system, reached errors of less than 4% for laminates up to 48 plies. The 144 virtual tests were conducted and analyzed in two days, compared to the one week required for the same experimental campaign. Since the compliance of this approach is demonstrated through physical testing, it must be robust enough to support the investigation of mechanical properties in different configurations, which are necessary for providing material characterization at the coupon level, the first level of the Building Block approach. Virtual testing is still far from replicating the stiffened panels with complex curvatures or thick laminates, which correspond to Level 3 (Sub component), in the Building Block certification methodology. However, virtual testing is expected to be a valuable tool to speed up the certification process for complex structures, which the aerospace industry seeks [66].

The civil aviation industry dominates the market for composite aircraft components. The industry is still experiencing supply chain shortages caused by the pandemic. Consequently, there is pressure on the industry to increase production rates by transitioning to an automated fiber placement (AFP) process that can handle larger material widths, thereby producing significant composite components. The push to become carbon-neutral will lead to the development of new propulsion technologies, which in turn will require new aircraft structural designs [68,69]. Although thermoplastic composites require less energy and can be manufactured at higher rates, thermosetting composites dominate the aviation industry due to their maturity. They allow for complex geometries and structures and can be implemented relatively easily. However, the lack of automation and the need for eight-hour curing times prevent high production rates [64]. A new generation of “fast-cure” thermosetting composites under development is expected to increase production rates [70].

Manufacturing developments in the aerospace sector promote efficiency and quality by investing in automation, such as automated tape layup (ATL) and automated fiber placement (AFP). These are suited to embrace the information technologies to their fullest extent, linking predictive models with manufacturing parameters. As discussed in [71], neural network-based inverse predictive models can generate a virtual sample and identify the required input conditions for producing a composite with the desired properties in automated manufacturing processes. The primary issue is the lack of data for training and assessing the models, which will be addressed in the coming years. Although CFRPs are increasingly replacing traditional metals in aircraft structures due to their superior strength-to-weight ratios, improving fuel efficiency, and reducing emissions, there is a need for new solutions to help airlines achieve their commitment to net-zero carbon by 2050. The International Air Transport Association (IATA) members passed this resolution at the 77th IATA Annual General Meeting in Boston, USA, from 3–5 October 2021.

**Figure 7:**
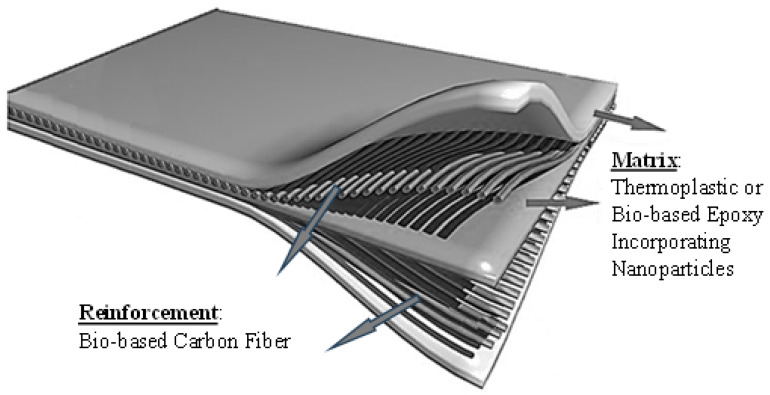
Next-generation composites.

In this sense, we envision two approaches to achieving sustainability in CFRPs: further development of thermoplastic matrices reinforced with bio-based carbon fibers or bio-based thermosetting epoxy resins reinforced with bio-based carbon fibers, as illustrated in Figure 7. The former option involves new production techniques and offers the potential for high production rates and assured recyclability. The latter takes advantage of a more mature production technology, speeding up its implementation. However, the recyclability issue with the thermosetting option remains critical, regardless of its origin; however, ongoing research is addressing this issue. The following describes some promising advances in technologies with the potential to be adopted by the aerospace industry.

### 7.1. Next-Generation Bio-Based Epoxy Resins

The modern aerospace industry benefits from well-established technology for carbon-reinforced composites, which has evolved from epoxy system developments over the past few decades. These systems strike a balance between cost, performance, and processability, ensuring the carbon fibers’ good adhesion to the matrix. The large-scale production of composite materials relies on petroleum-based epoxy monomers, most of which are derived from bisphenol A (BPA) [72]. The National Institute of Environmental Health Sciences (NIEHS) and other health regulatory bodies consider BPA an endocrine-disrupting chemical [73]. These factors and concerns about sustainability drive the need to investigate renewable, less toxic feedstocks for bio-based formulations.

Bio-based epoxy resins are produced using raw materials such as vegetable oils, lignin, rosin, and other plant-based substances. While extensive research has been conducted into developing high-performance, sustainable resin systems, few studies have examined their potential for large-scale composite production [74]. There are many bio-based epoxies, several of which are high-performance materials suitable for aerospace composites. The epoxy group is the most extensively researched of the biobased thermosets. However, two questions remain unanswered: how can they be produced on a large scale at an affordable cost, and how effectively can they be integrated into composites [72]?

New studies disclosed novel approaches to the chemical recycling of bio-based resins. One approach is to depolymerize a bio-based polyester thermoset based on succinic acid and epoxidized linseed oil. This chemical recycling enables the recovery of raw compounds [75]. Another bio-based, degradable, hyperbranched epoxy resin (EFTH-n, n = 3, 6, 9, 12) was found to improve the toughness, strength, modulus, and elongation of DGEBA, achieving the highest mechanical properties with the 12 wt% EFTH-6/DGEBA composite. In an environmentally friendly phosphoric acid solution, the formulation degraded by 99.8% [76]. Another relevant approach is the incorporation of additional functionalities, as demonstrated by synthesizing an intrinsically flame-retardant epoxy resin using a green, bio-based molecular design based on eugenol. This approach provided optimal flame retardancy and mechanical performance while maintaining satisfactory mechanical strength. This epoxy system exhibits mechanical properties comparable to commercial bisphenol-A-type epoxy resins, with a glass transition temperature of 294.5 °C [77].

Although comparable mechanical characterization based on ASTM or ISO standards is needed, it is often disregarded. However, an experimental mechanical characterization following ASTM standards was conducted on four epoxy resin systems: one conventional system and three bio-epoxy systems, two of which were non-recyclable with varying levels of bio-content. The main conclusion was that the recyclable bio-epoxy resin produced the most promising results with 27% bio-content [78]. Another study of biocomposites in which natural fibers were incorporated into a high-performance epoxy resin containing 51% bio-based carbon followed ASTM standards for mechanical characterization. Key factors in the performance of hybrid biocomposites reinforced by natural fibers are the efficiency of mechanical stress transferred from the epoxy matrix to the natural fibers and the uniform dispersion of the natural fibers in the matrix. Once again, the authors emphasize the potential of biocomposites for use in the aerospace industry [18].

Still, the need to replace traditional epoxy resins fully drives research forward. The mechanical and thermal performance levels necessary to attain those of these conventional materials pose difficulties in this endeavor. A certain amount of oil-based content is essential to reach the required performance of bio-based epoxy resins. The commercialized bio-based epoxies reflect the need to limit the bio-content to between 28 and 43%, as pointed out by Terry and Taylor [79], to achieve similar performance to oil-based epoxies in terms of strength, stiffness, toughness, and glass transition temperature. Table 6 presents relevant commercialized bio-based epoxies, comparing their mechanical properties and glass transition temperature, $T_{g}$, with those of an exemplary oil-based resin.

Although some fully biobased epoxy resin systems produced from renewable phenolic compounds or, more recently, furan resins reached mechanical properties comparable to conventional oil-based epoxy resin systems, the quest for fully bio-based epoxies is far from over [81]. Its adoption in primary structural parts is not imminent despite all advances.

Recent news reported the research project (iLAuNCH), which joins three Australian universities (the University of Southern Queensland (UniSQ), the Australian National University, and the University of South Australia) and a private company, Change Climate Pty Ltd./BYOXY [82], is studying a novel bio-epoxy formulation claimed insensitive to UV radiation and complying with aerospace environments, in manufacturing and testing composite structures targeting the aerospace industry. BYOXY is a 100% bio-based epoxy resin certified free of Bisphenol A. Laboratory experiments at UniSQ have shown that the bio-epoxy resin system exhibits very low shrinkage and excellent UV resistance. Further work will thoroughly assess resistance to radiation, atomic oxygen, corrosion, outgassing, and thermal loading [83]. However, no results have been reported to evaluate these claims independently.

### 7.2. Next-Generation Bio-Based Carbon Fiber

Carbon fibers are produced by the controlled pyrolysis of organic precursors, which become infusible when cross-linked, typically polyacrylonitrile or pitch. These precursors are heated in an inert atmosphere to temperatures between 1200 °C and 3000 °C to virtually drive off the inorganic components, thereby converting them into carbon fiber. There are four types of carbon fibers: high modulus (>300 GPa), intermediate modulus (>200 GPa), low modulus (100 GPa), and high strength (>4 GPa), which are defined by the heat-treatment temperature. Polyacrylonitrile fibers dominate the industry, as almost all commercial carbon fibers employ polyacrylonitrile as a precursor [84]. In the 1930s, the “Sohio Acrylonitrile Process” emerged as an innovative, single-step method of production, making acrylonitrile readily available as a key raw material for chemical manufacturing worldwide. Being inexpensive and high-purity is responsible for the exponential growth of the acrylic plastics and fibers industries.

Until today, acrylonitrile industrial production is carried out via the Sohio process, derived from oil [85]. Acrylonitrile is now an essential commodity chemical. It is used to produce polyacrylonitrile, from which carbon fibers are made. The need to find more sustainable ways to produce carbon fibers, which are excellent reinforcements for composite structures in the aerospace industry, led to the search for new methods that do not rely on petrochemicals. The other oil-based precursor used for carbon fiber production is pitch, derived from asphalt, coal tar, or polycyclic aromatic hydrocarbons derived from polyvinyl chloride. It is the second most crucial feedstock, after polyacrylonitrile, for producing carbon fibers. These fibers have superior mechanical properties, with the highest carbon yield (80–90%).

A recent approach proposed asphaltene as a potential raw material for carbon fiber production, due to its high carbon concentration and low cost. Asphaltene is the heavy fraction of bitumen and crude oil removed during refining, considered a low-value by-product or waste. Research into processing technology is still in the early stages, although some progress has been reported [86].

The bio-based precursors are divided into lignin and viscose. Lignin is a natural substance extracted from biomass and found in lignocellulose. It is sometimes referred to as ‘technical lignin’. Some of the significant lignin bio-sources are Jute, Banana, Coir, Flax, Maize, Sisal, Wood, Bagasse, Rice husk, with contents of lignin between 5% (jute) to 45% (coir) [87].

Viscose, sometimes called rayon, is a semi-synthetic fiber chemically processed from cellulose obtained from wood pulp, pine, spruce, hemlock trees, and cotton linters. Even though the cost of raw materials is lower for rayon-based fibers, the carbon yield is lower, meaning only a low percentage of the original material is converted into carbon fibers (10–25%), leads to higher production costs than the traditional production method, limiting the amount that can be commercialized. Moreover, the mechanical properties of the carbon fibres produced from rayon reach lower tensile modulus and strength than those produced by conventional methods, preventing their use in structural composites [88].

Table 7 compares the different precursor types used to produce carbon fibers. Briefly, the advantages of oil-based materials are their superior mechanical properties, which are critical for highly demanding sectors in terms of mechanical performance. Aerospace composites require carbon fibres with a tensile strength of over 3 GPa and a tensile modulus of 450 to 940 GPa [89].

Table 8 shows some results from the blending strategy to improve the final mechanical properties. Blending the lignin with the synthetic oil-based polymers, such as polyacrylonitrile (PAN), poly(ethylene oxide) (PEO), polythylene terephthalate (PET), polyvinyl Alcohol (PVA), and thermoplastic elastomer polyurethane (TPU). When PAN is blended with either lignin or cellulose to improve mechanical performance, reduce costs, and increase sustainability, the resulting blend displays incompatibility between the PAN and these materials. This incompatibility results in defects and voids that harm the mechanical properties. A blend of fully renewable cellulose and lignin avoids these disadvantages and is a promising alternative. Nevertheless, these emerging carbon fibres are still far from meeting the requirements of the aerospace sector [91].

Recently, acrylonitrile has been successfully synthesized from sustainable feedstocks. As pointed out, the full-scale process estimation of the selling price of acrylonitrile from lignocellulosic sugars and sucrose is in the range of fossil fuel-derived acrylonitrile prices. Another benefit is the reduction of 14% of greenhouse gas emissions with this process compared with the traditional one [93,94].

Recently, Airbus’ researchers used a carbon fiber derived from a bio-based acrylonitrile, not specified, to manufacture a proof-of-concept nose panel for Airbus Helicopters’ H145 Pioneer Lab. Airbus stated that the panel had stiffness and strength comparable to the conventional part and was flight-tested in May 2024. The primary reason for choosing this alternative fibre to demonstrate its airworthiness was its small size, which made it easy to produce rapidly. Since it is non-structural, it is a convenient part of the aircraft for testing the material [95].

### 7.3. Next-Generation Thermoplastic Composites

Several financed projects aim to develop aircraft parts and panels for the empennage and fuselage made of thermoplastics reinforced with carbon fibers. An assessment study consistently ranked thermoplastic CFRP panels as the most sustainable option, considering environmental factors, costs, and performance, compared with aluminum and traditional thermosetting CFRP solutions [96].

The aerospace industry has been extensively using semi-crystalline, high-performance thermoplastic polymers such as polyether ketone ketone (PEKK), polyetheretherketone (PEEK), polyphenylene sulfide (PPS), and low-melt polyaryletherketone (LM-PAEK). The amorphous polymer Polyetherimide (PEI) has also been used due to its high performance. The mechanical properties of these polymers are comparable to, if not superior to, those of the epoxy resins used in aerospace, as shown in Table 9. Their main advantage, besides their recyclability, is their welding capacity, which leads to weight reduction by eliminating the need for fasteners and contributing to the reduction of associated stress concentration issues. The same fusion bonding technique can be used to repair thermoplastic composites without surface preparation. Induction, resistance, and ultrasonic welding are the most promising techniques [97].

A recent systematic review establishes the current state of affairs and examines future developments in thermoplastics reinforced with carbon fibers. These materials offer superior structural performance and can easily be tailored to the load direction [98]. The aerospace sector reports notable advancements without disclosing the technical details about the manufacturing process or the experimental testing conducted to prove the concept. Although this is understandable, it prevents the research community from sharing these advancements.

There are still few results available in the literature for PEEK/PEKK welded joints, and no studies have comprehensively investigated fatigue under different environmental conditions. Regarding the static tensile lap shear test, results depend on the number of plies and the orientation of the layup. Some fatigue studies have also been conducted at room temperature. While the impact of environmental conditions on static strength has been studied, its effect on fatigue lifetime has not. For the static response, lap shear strength ranges from 29 to almost 53 MPa for both PEKK and PEEK. There is little difference in strength between single-lap and double-lap joints as shown in Table 10.

The fatigue behaviour follows the typical S-N curve, whereby the fatigue strength decreases with the number of cycles. In Table 11, the S-N experimental curves can be explained using the logarithmic function LSS (N) = a log N + b, with a correlation coefficient higher than 0.8. The LSS decays by between 30% and 40% after 1 million cycles and by between 10% and 27% after 10 million cycles.

A few studies in the literature report on the effect of environmental conditions on static lap shear strength. Two cases are presented for CF/PEKK and CF/PEEK in Table 12 and Table 13, respectively. The case in Table 13 demonstrates that incorporating 5% aluminium nitride by weight improves joint performance and enables the material to withstand harsh environmental conditions. However, the conditioning time was half that of the case in Table 12.

Collaborations between Airbus and Boeing, along with consortia such as Clean Sky 2 and NASA’s HiCAM (Hi-Rate Composite Aircraft Manufacturing) program, demonstrate their long-term commitment to thermoplastics. Airbus recently unveiled a new fuselage design as part of the Clean Sky 2 initiative. The 8-m-long, 4-m-wide Multifunctional Fuselage Demonstrator (MFFD), primarily composed of thermoplastic composites reinforced with carbon fiber, will undergo testing to assess the potential of these materials for structural applications. Developed under the Large Passenger Aircraft platform of Clean Sky 2, the MFFD aims to accelerate aircraft production, thereby enhancing the competitiveness of the European aerospace industry. The new fuselage design is lighter, as it reduces the need for fasteners while accommodating all necessary electrical, mechanical, pneumatic, and hydraulic systems, thereby improving production efficiency [103,104].

### 7.4. Further Paths for Innovation

Innovations in nanotechnology are expanding the boundaries of material performance. Integrating carbon nanotubes and graphene into composite structures enhances their mechanical strength, thermal stability, and electrical conductivity. These nano-reinforced materials are promising for applications requiring extreme durability and minimal weight, such as critical structural elements and high-performance engine components. Recently, the selective placement of an electrospun polyamide nanofiber veil at the midplane of the laminated composite has provided an effective means of interlaminar toughening without compromising its in-plane mechanical properties or significantly increasing its thickness or weight [105,106]. Leading also to commercial solutions of advanced composites with considerable improvement of the mode I and II fracture toughness and fatigue life [107]. More recently, extensive fatigue tests of carbon fiber and glass fiber laminates and hybrid laminates proved the effective improvement of the fatigue life after incorporating electrospun polyamide nanofiber interleaving veils [108,109]. However, challenges remain in scaling these materials for broader use, particularly in ensuring their compatibility with existing manufacturing processes. Nonetheless, the potential for nano-engineered composites to reduce weight and improve lifespan aligns well with the industry’s sustainability goals [110,111].

Another innovation shaping the future of aerospace materials is additive manufacturing, also known as 3D printing. This technique enables the precise production of complex geometries, reducing waste and optimizing material usage. It is particularly advantageous for the aerospace industry, where weight savings are critical. Building parts layer by layer allows for intricate designs that would be challenging to achieve with traditional manufacturing methods. Materials such as titanium and high-strength aluminum alloys are commonly used in additive manufacturing for aerospace applications, contributing to both lightweight construction and material efficiency. Furthermore, it also supports rapid prototyping and on-demand production, which can streamline the supply chain and reduce the industry’s overall environmental footprint. The future of aerospace materials lies in integrating advanced composites, nanotechnology, renewable bio-composites, and additive manufacturing [110,111,112].

## 8. Conclusions

The aerospace industry is transforming, driven by the growing demand for more sustainable and efficient materials. This shift is crucial as manufacturers strive to reduce fuel consumption, minimize environmental impact, and prolong the service life of aircraft components. Advanced composite materials, particularly lightweight composites like carbon fiber-reinforced polymers (CFRPs), play a central role in this evolution. CFRPs offer a high strength-to-weight ratio, which directly contributes to improved fuel efficiency and reduced emissions. These composites are stronger and lighter than traditional metallic materials, exhibiting excellent resistance to fatigue and environmental degradation. It allows for longer service intervals and fewer replacements [110,111]. CFRPs continue to represent a core structural material in the aerospace industry due to their high strength-to-weight ratio, fatigue resistance, and extensive lifecycle benefits, including improved fuel efficiency and reduced emissions. The solutions adopted in the primary structures of large aircraft, such as the Boeing 787 and the Airbus A350, in load-bearing components enable these large aircraft to maintain their structural integrity while significantly reducing overall weight, thereby improving efficiency and range. The aircraft’s operational efficiency and lower environmental impact serve as a model for future aerospace designs, where composite materials are expected to play an even greater role. Continued innovations in composite manufacturing, lifecycle assessment, and structural integrity are expanding the potential applications of CFRPs and related materials, underscoring their role in the aerospace industry’s ongoing evolution toward sustainable, high-performance solutions. Nonetheless, CFRP production remains energy-intensive, offsetting some of the environmental gains of a more efficient aircraft. Efforts to improve the manufacturing process are ongoing to reduce the offset.

The competitive landscape between major aerospace companies, particularly Boeing and Airbus, has further accelerated the development of material innovations. The rivalry between these giants has driven continuous advancements as each strives to outdo the other in terms of efficiency, cost reduction, and environmental performance. Boeing, for example, has focused on open innovation to integrate external expertise and advance its material technologies, while Airbus has explored bio-composites and new types of lightweight materials to strengthen its sustainability profile. This competition encourages each company to pursue sustainability goals and drives the industry to adopt advanced materials and efficient manufacturing processes [112].

Bio-composites, recycled carbon fiber composites, and thermoplastics were thoroughly analysed, with their advantages and disadvantages exposed, as well as some manufacturing and implementation challenges. Each of these materials plays a specific role in an increasingly sustainable aviation industry, and all face challenges in their deployment. However, these materials lack the mechanical properties for structural and critical applications. This way, these materials are primarily used in secondary and interior structures. Challenges in the manufacturing processes and implementation of these materials, such as chemical treatments and surface modifications, as well as their properties, including flammability and moisture sensitivity, remain significant barriers.

Overall, recycled carbon fibre composites are a valuable and sustainable material for aircraft, particularly for non-critical components. While issues regarding fibre length, contamination, and cost remain, developments in solvolysis and electrodynamic fragmentation are paving the way for broader acceptance. To realise the environmental and financial advantages of rCF recycling and processing within the aerospace industry, constant innovation in these fields will be essential.

On the same note, thermoplastics such as PEEK and PPS, while having better mechanical properties, making them more valuable for structural components, require a significant amount of energy in their manufacturing processes, making them environmentally and economically more challenging. Thermoplastics are advantageous in applications demanding damage tolerance and environmental resilience, with PPS, PEEK, and PEKK proving effective in high-temperature and corrosive environments. These materials can be reheated, reshaped, and welded, enabling repairs that are not feasible with thermosets. This property is particularly beneficial in the aerospace industry, where maintenance efficiency is crucial. Therefore, thermoplastic composites represent a promising category of materials in aerospace due to their durability, recyclability, and reduced processing times. Continued advancements in manufacturing technologies, such as automated layup processes and enhanced welding techniques, are anticipated to expand the application of thermoplastic composites in intricate aerospace structures.

The aerospace sector’s shift toward sustainable materials aligns with global sustainability pledges. As the industry strives to achieve high environmental standards, developing robust environmental assessment and lifecycle management frameworks will be crucial in evaluating novel materials.

In conclusion, all these different materials offer promising avenues for a more sustainable aerospace industry, though their adoption requires addressing specific technical, economic, and regulatory challenges. Material engineers and researchers are continuously working to improve both the manufacturing and recycling processes for producing parts with new materials. Recycling used materials is a more efficient process throughout the entire life of the part.

## Figures and Tables

**Table 1:** Summary of three-point bending test results for different composite materials.

Composite	*σ* (MPa)	$E_{b}$ (GPa)	Ref.
CFRP	760	57.1	[22]
CFRP	583	17.2	[23]
Kevlar	302	11.7	
GFRP	131	7.64	[24]
Glass Plain Weave	116	4.5	[25]
Twill Glass	380	12.6	
Flax 200	179	2.7	
Flax 200 Dry	245	4.5	
Flax 300	179	2.3	
Flax 300 Dry	190	2.8	
Flax—RT air	169	11.4	[26]
Flax—60 °C air	164	11.7	
Flax—100% RH	127	6.2	
Flax—60 °C water	60.1	2.1	

**Table 2:** Comparison of tensile and flexural (3PB) properties of virgin carbon fibre (vCF) and recycled carbon fibre (rCF) with unidirectional (UD) and plain woven (PW) fibre architectures.

Fibre Architecture		Tensile Modulus (GPa)	Tensile Strength (MPa)	Flexural Modulus (GPa)	Flexural Strength (MPa)
Virgin CFRP	UD	226.6	3485	89.77	1030
	PW	208.8	2815	39.45	539.2
Recycled CFRP	UD	214.5	2918	64.45	999.9
	PW	189.6	2341	26.95	595.8

UD—Unidirectional; Plain woven—PW

**Table 3:** Material mechanical properties and their suitability.

Material	Fatigue Resistance	Impact Resistance	Application Suitability
Aluminum Alloys	Moderate	Moderate	Load-bearing
CFRP’s	Very High	High	Load-bearing
Thermoplastic Composites	High	High	Non-load-bearing

**Table 4:** Mechanical Properties Comparison.

Material	Density (g/cm^3^)	Strength to-Weight Ratio	Recyclability	Common Applications	Thermal Resistance
Aluminum Alloys	2.7	Medium	High	Fuselages, Wings	Moderate (<300 °C)
Titanium Alloys	4.5	High	Moderate	Engine Casings, Landing Gear	High (600 °C)
CFRP’s	1.6	Very High	Medium	Fuselage wings, Empennage	Moderate (350 °C)
Thermoplastic Composites	1.6	High	Medium	Interiors, Secondary Structures	Moderate (250 °C)

**Table 5:** Cost and economic trade-offs comparison.

Material	Initial Production Cost	Maintenance Cost	Repairability	Lifecycle Cost Efficiency
Aluminum Alloys	Low	Low	Easy	Medium
CFRP’s	High	Moderate	Difficult	High (Long-Term)
Thermoplastic Composites	Medium	Low	Easy	Medium-High

**Table 6:** Mechanical properties of commercial bio-epoxy resins and one exemplary oil-based epoxy resin (Adapted from [80]).

Name	Tensile Strength (MPa)	Tensile Modulus (GPa)	Flexural Strength (MPa)	Flexural Modulus (GPa)	Tg (°C)	Epoxy System	Feedstock Nature	Bio Content (%)	Manufacturer
AMB	40	1.9	70	2.1	47	AMPRO BIO/ Slow Hardener	Cashew nut shell liquid	40–60	Gurit AG, Zurich, Switzerland
EVO	72	3.4	120	3.1	86	SR Surf Clear EVO/ SD EVO Fast	Vegetable oil	40	Sicomin, Châteauneuf-les-Martigues, France
SR56 + SD	74	3.7	128	3.6	76	SRGreenpoxy56/ SZ8525	Plant origin	35–41	Sicomin
SP100	57	2.6	81	2.3	47	SuperSap 100/ 1000 Hardener	Waste pine and vegetable oils	37	Entropy, San Francisco Bay Area, CA, USA
F2501	69	3.1	113	2.8	100	FORMULITE 2501A/ FORMULITE 2401B	Food waste origin: cardanol	34	Cardolite Corp., Bristol, PA, USA
BP36	58	2.8	97	2.9	54	BioPoxy 36/ Clear Hardener	Soybean, cashew nut oil and recycled egg shells	32	Ecopoxy, Winnipeg, MB, Canada
IB2	68	3	109	3.1	71	IB2/ Amine Hardener	Plant origin: glycerol	38	Easy Composites, Stoke-on-Trent, UK
INF810	71	3.2	116	3.3	69	INF810 Infugreen810/ SD8822	Plant origin	38	Sicomin
LY556 + HY917	88	3.1	150	3.2	133	Araldite LY556/ Aradur 917	Oil based	0	Huntsman Corp., Freeport, TX, USA

**Table 7:** Cost, Greenhouse gas (GHG) emissions, and mechanical properties of various carbon fibers depending on the precursor type used (Adapted from [89,90]).

Precursor type		Cost ($/kg CF)	GHG emissions (kgCO_2_eq/kg CF)	Tensile Strength (GPa)	Elastic Modulus (GPa)	Density (g/cm^3^)	Diameter (mm)
Oil-based Carbon Fibers	Polyacrylonitrile (PAN)	11–260	20–30	3–6.8	170–940	1.78–1.98	5–10
	Mesophase pitch	>40	—	1.83–3.80	133–935	1.8–2.2	10–11
	Asphaltenes-Isotropic pitch	6.64	6–17	1.13	71		
Bio-based Carbon Fibers	Lignin	5–11	5–15	0.52–1.07	28.6–82.7	1.4–1.5	5–10
	Rayon (Viscose)	—	—	0.6–1.7	29–65	1.8	1–55

**Table 8:** Comparison of the different blended precursor types and the properties of the carbon fibers (Adapted from [92]).

Precursor type	Carbon Content (%)	Degree of Crystallinity (%)	Tensile Strength (GPa)	Tensile Modulus (GPa)
Softwood kraft lignin	55.1		1.06 ± 0.07	52 ± 2
Birch wood lignin	63.7		0.66	40.7 ± 6.3
Organosolv hardwood lignin	64.3		0.355 ± 0.053	39.1 ± 13.3
Hardwood kraft lignin	58.5		0.52 ± 0.182	28.6 ± 3.2
Acetylated softwood kraft lignin	61.3–62.8		1.06 ± 0.07	52 ± 2
Softwood/hardwood kraft lignin	63.8		0.233–0.377	25–33
Switchgrass/ boxwood lignin	60.3		0.23–0.75	30.4–41.8
Lignin (25%)/ PAN blend	65.1		2.25	217
Lignin (30%)/ PAN blend	64.5		1.72 ± 0.2	230 ± 7
Hardwood kraft lignin/PEO	57.3–59.7		0.458 ± 0.097	59 ± 8
Lignin/ PEO (97–3)	49.3		0.448 ± 0.070	51 ± 13
Lignin/ PET (75–25)			0.703	94
Lignin/ PVA (70–30)			0.351 ± 0.108	44.5 ± 9.6
Lignin/ TUP (50–50)			1.10 ± 0.100	80 ± 10
PAN/ nanocellulose crystals			1.80–2.30	220–265
Cellulose/ lignin (30–70)			1.07	76
Cellulose/lignin (50–50)			0.710–0.920	46–51

**Table 9:** Mechanical properties provided by the manufacturers.

		Epoxy Resin	Thermoplastics				
	ASTM	Hexcel^®^ HexFlow^®^ RTM 6	PEEK *	PEKK *	PPS *	LM-PAEK (AvaSpire^®^ AV-722)	Ensinger TECAPEI™ natural PEI
Density (g/cm^3^)	D792	1.14	1.34	1.28	1.34	1.32	1.27
Tensile Modulus (GPa)	D638	2.89	3.88	3.61	4.2	3.7	2.96
Tensile Strength (MPa)	D638	75	99	76.2	116	89	121
Elongation at Break (%)	D638	3.4	26	13.4	15.7	25	40
Flexural Modulus (GPa)	D790	3.3	4.3	3.37	4.15	3.7	3.31
Flexural Strength (MPa)	D790	132	156	124	129	141	159

* Averaged properties of PEEK, PEKK and PPS provided by “https://www.matweb.com/”.

**Table 10:** Comparison of static lap shear strength (LSS) for CF/PEKK and CF/PEEK.

Material	LSS (MPa)	Type	Reference
CF/PEKK	33.2	SLJ	[99]
CF/PEKK	29.5	SLJ	[100]
CF/PEKK	31.2	SLJ	[101]
CF/PEKK	52.2	SLJ	[102]
CF/PEKK	48.9	DLJ	[102]
CF/PEEK	49.8	SLJ	[102]
CF/PEEK	52.9	DLJ	[102]

SLJ—Single-Lap Joint; DLJ—Double-Lap Joint.

**Table 11:** Results of regression trend analysis for the S-N curve after fitting a logarithmic function, based on experimental data from [99,102].

Cycles to Failure	CF/PEEK [102]	CF/PEKK [102]			CF/PEKK [99]
	DLS	SLS	DLS	SLS	SLS
		Shear Stress (MPa)			
500	81	92	68	68	22.0
1000	77	87	64	64	20.7
10,000	62	69	53	50	16.1
100,000	48	52	42	37	11.6
1,000,000	33	34	30	23	7.0
10,000,000	19	17	19	9	2.5

DLS: double lap shear; SLS: single lap shear.

**Table 12:** Single-lap strength experimental results of CF/PEKK at different conditioning and testing temperatures [101].

Environmental Conditioning	Testing Temperature	LSS (MPa)
-	25 °C	31.2
-	100 °C	26.3
-	120 °C	21.5
-	140 °C	16.3
-	160 °C	7.4
-	180 °C	4.6
85 °C/85% —40 days	25 °C	16.5
85 °C/85%—40 days	100 °C	13.9
85 °C/85%—40 days	120 °C	12.9
85 °C/85%—40 days	140 °C	8.8
85 °C/85%—40 days	160 °C	3.6
85 °C/85%—40 days	180 °C	1.9

**Table 13:** Single-lap strength experimental results of CF/PEEK (with 5 wt.% AlN ) at different conditioning and testing temperatures [100].

Environmental Conditioning	Testing Temperatures (°C)	LSS (MPa)
25°C/50% RH	−30	30.2
25°C/50% RH	25	29.5
25°C/50% RH	85	28.7
25°C/50% RH	145	24.1
85°C/90% RH—28 days	−30	29.7
85°C/90% RH—28 days	25	28.6
85°C/90% RH—28 days	85	26.5
85°C/90% RH—28 days	145	21.6

## Data Availability

Not applicable.

## Abbreviations

The following abbreviations are used in this manuscript:

CFRP	Carbon Fiber-Reinforced Polymer
CF	Carbon Fiber
TPC	Thermoplastic Composite
LCA	Life Cycle Assessment
FAA	Federal Aviation Administration
EASA	European Union Aviation Safety Agency
OEM	Original Equipment Manufacturer
SA	Sustainable Aviation
